# Exposure to air pollution and hospitalization due to COVID-19 in São José dos Campos, Brazil

**DOI:** 10.1590/1414-431X2021e12273

**Published:** 2022-11-11

**Authors:** A.O.R. Santos, B.R. Lucarevschi, C.J.D. Cunha, P.C. Ribeiro, A.C.G. Cesar, L.F. Nascimento

**Affiliations:** 1 Departamento de Medicina, Universidade de Taubaté, Taubaté, SP, Brasil; 2 Faculdade de Engenharia de Guaratinguetá, Universidade Estadual Paulista Júlio de Mesquita Filho, Guaratinguetá, SP, Brasil; 3 Instituto Federal de Educação, Ciência e Tecnologia de São Paulo, Bragança Paulista, SP, Brasil; 4 Programa de Pós-graduação em Ciências Ambientais, Universidade de Taubaté, Taubaté, SP, Brasil

**Keywords:** COVID-19, Coronavirus, Air pollution, Nitrogen dioxide, Ozone

## Abstract

The association between exposure to air pollutants and respiratory diseases is well known. This study aimed to identify the association between this exposure and hospitalizations for COVID-19 in São José dos Campos, SP, a medium-sized city, between April 2020 and April 2021. Hospitalization data, concerning code B34.2, was supplied by DATASUS, and data concerning pollutants and climate variables were supplied by CETESB. Cases were quantified by sex, age, length of hospital stay in days, and type of discharge, whether hospital discharge or death. The negative binomial regression model was chosen. Estimates were produced for the relative risk (RR) of significant exposure to pollutants (P≤0.05) with a 10 µg/m^3^ increase of pollutant, as well as for excess hospitalizations. There were 1873 hospitalizations, with a daily average of 4.7 (±3.8), ranging from zero to 21: 716 deaths (38.2%) were recorded, 1065 admissions were men, and women were less susceptible (OR=0.82). The average age of women was higher than that of men; in cases of death, men were older than women; discharged patients were younger. All the above variables were significant. The risk of ozone exposure was higher and more significant in Lag 2, and the risk of nitrogen dioxide exposure was high in Lag 3, which was the period of the highest increase in hospitalizations, at 11.3%. The findings of this study, the first conducted in Brazil, corroborate the results of studies conducted in other centers.

## Introduction

The disease caused by the new coronavirus (COVID-19) was first detected in Wuhan, China, in December 2019, and declared a global pandemic by the World Health Organization (WHO) (1). COVID-19 is a highly transmissible and fatal disease caused by the new coronavirus, SARS-CoV-2, and in general, infected patients show mild to moderate symptoms including sore throat, fever, shortness of breath, dry cough, loss of smell and taste, while some critical patients present with pneumonia, severe acute respiratory syndrome (SARS), renal failure, and death (2,3).

Epidemiological studies have demonstrated associations between exposure to air pollutants and morbidity and mortality from respiratory diseases, since the lungs are constantly exposed to these contaminants and may result in the development and exacerbation of lung diseases such as laryngitis, tracheitis, pneumonia, chronic obstructive pulmonary disease (COPD), asthma, and lung cancer (4-11). Recent studies have demonstrated the correlation between short-term and chronic exposure to environmental air pollution and infection by COVID-19 (12-15).

According to the WHO, air pollutants most widely studied are ozone (O_3_), the airborne particulate matter with an aerodynamic diameter of less than 10 µ (PM_10_), carbon monoxide (CO), sulfur dioxide (SO_2_), and nitrogen dioxide (NO_2_) (16).

This study aimed to identify possible associations between exposure to air pollutants and hospitalizations for COVID-19 in residents of São José dos Campos, Brazil.

## Material and Methods

An ecological time-series study was developed using data from hospitalizations for COVID-19 in residents of São José dos Campos, SP, between April 1, 2020 and April 30, 2021.

### Study location

São José dos Campos is a municipality in the interior of the state of São Paulo, located about 90 km from the state capital (23°10′′47′′S, 45°53′14′′W). Important highways connect the São José dos Campos area: on the north by Highway SP-50 (south of Minas Gerais) and Campos do Jordão; on the south by Tamoios Highway (SP-99), Paulista North Coast, and Carvalho Pinto Highway (SP-70); and in the east-west direction by BR-116 (President Dutra Highway). All these highways have a heavy traffic flow.

In 2017, São José had an estimated population of 700 thousand inhabitants and in 2016 it had an estimated fleet of 400 thousand vehicles (17).

Agriculture flourished during the 19th century, but industrial development in the city only expanded in the second half of the 20th century, providing a spark for the technology sector. Important companies such as General Motors, Petrobras, and Embraer, among others, have based their head offices within the municipality's jurisdiction. It is also home to renowned educational and research centers such as INPE (National Institute for Space Research), UNESP (São Paulo State University), ITA (Aeronautical Technological Institute), FATEC (State Technology College), and UNIVAP (Paraíba Valley University), among others. The city is an important technological center for military and metallurgical equipment and hosts the headquarters of the largest aerospace complex in Latin America. In addition to traffic, which consists mainly of heavy vehicles and busses operating on the highways and within the city limits (in urban areas), the automotive and petrochemical industries, along with all the other factors mentioned above, are sources of air pollution that contribute to the morbidity and early mortality of the local population.

### Material and Methods

Hospitalization data was provided by DATASUS (18), following the SUS (Brazilian public health system) Hospital Information System (SIHSUS) using the diagnosis code B34.2 (ICD-10), including hospitalization date, length of stay in days, sex, and type of discharge (hospital discharge or death).

The concentrations of pollutants PM_10_, NO_2_, and O_3_ were provided by the State of São Paulo Environmental Company (CETESB) (19). Daily data on environmental temperatures and relative air humidity were also provided by CETESB.

Given that the data used for hospitalization were count data, we chose the negative binomial regression model to avoid overdispersion, which can occur when the variance of the dependent variable is greater than the average of this variable.

Comparison of the average length of stay and age according to sex, and length of stay and age according to type of discharge, i.e., hospital discharge or death, was done using the Student's *t*-test. The odds ratio (OR) was estimated between the variables sex and type of discharge, and the level of association between these variables was identified using the chi-squared test.

The three pollutants, temperature, and air humidity, controlled for day of the week and by calendar day, were entered in the negative binomial regression model. The β coefficients provided by the negative binomial regression were transformed into relative risk (RR) using the formula RR = exp (β × conc_pol), where conc_pol is the pollutant concentration. The excess hospitalization was estimated as an increase in percentage (IP), based on a 10 µg/m^3^ increase of the pollutant, using the formula IP = (exp(β × 10) - 1) × 100.

The analyses were conducted using the Stata v10 program (USA), with an alpha of 5% as the significance level. Since the analyzed data was anonymous, there was no requirement to submit the study to the Research Ethics Committee.

## Results

There were 1873 COVID-19 cases hospitalized during the study period, with a daily average of 4.7 (±3.8), ranging from a daily minimum of zero to a daily maximum of 21 hospitalizations; 716 (38.2%) deaths were recorded. The hospitalization rate during the study period was 254.14 cases/100,000 inhabitants.

There were 1065 male (56.9%) and 808 female hospitalizations, and the distribution of deaths by sex was 428 males (59.8%) and 288 females. The chi-squared test showed a significant association between the variables (P=0.045) with OR=0.82 (95%CI: 0.62-0.99) indicating that female sex may be a protective factor.

The average age (in years) was 61.4 for women and 59.6 for men (P=0.015). For case fatalities, the average age was 66.6 for men and 56.5 for women (P<0.001). The average age for case fatality was 66.6 (±13.9) and the average age for patients discharged from the hospital was 56.5 (±16.0) (P<0.01).

The length of stay was significantly longer (P<0.001) for case fatalities (12.6 days) than for cases that were discharged (8.1 days). No significant difference was found between sexes concerning length of stay, with an average of 9.9 days recorded for men and 9.6 days for women.

The average, standard deviations, and minimum and maximum values for concentration of pollutants and climatic variables are shown in Table 1. The values for pollutant concentrations remained unchanged from the 2019 values.

**Table 1:** Descriptive analysis of climate variables and pollutant concentrations in São José dos Campos, 2020-2021.

	Mean (SD)	Min-Max
Ozone (μg/m^3^)	57.3 (21.5)	9-99
Nitrogen dioxide (μg/m^3^)	37.5 (16.3)	7-108
Particulate matter (PM_10_) (μg/m^3^)	21.0 (10.0)	5-77
Temperature (°C)	28.4 (4.2)	14.6-38.7
Relative Humidity (%)	43.6 (13.3)	16-89

Data are reported as means and standard deviations (SD) and minimum and maximum values.

**Figure 1:**
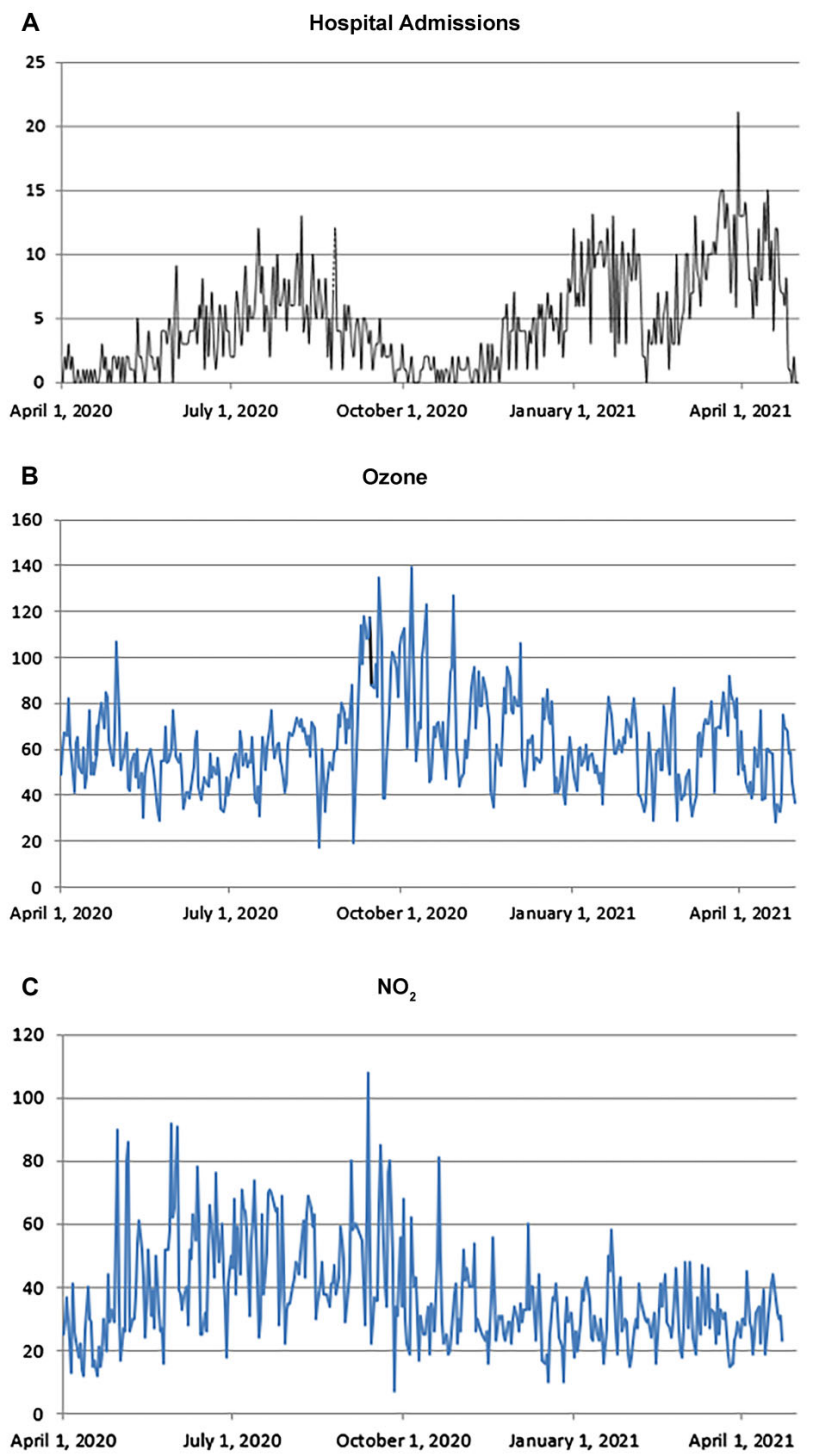
Daily data on (**A**) hospital admissions due to COVID-19 and (**B**) ozone and (**C**) nitrogen dioxide (NO_2_) concentrations (ug/m^3^) in São José dos Campos, Brazil in 2020-2021.

The daily distribution of cases and O_3_ and NO_2_ concentrations during the 13-month period is shown in Figure 1A, B, and C.

The coefficients and respective standard errors for concentration of pollutants according to lags of zero to seven days (Lag 0-Lag 7) are shown in Table 2. No association was found between exposure to PM_10_ and hospitalization. The highest RR for O_3_ was shown for exposures occurring two days before hospitalization (Lag 2), with RR=1.006, while the highest relative risk for NO_2_ was in Lag 3 with RR=1.012. The RR for a 10 µg/m^3^ increase in pollutant concentration was RR=1.061 (95%CI: 1.016-1.108) for O_3_ and RR=1.125 (95%CI: 1.050-1.205) for NO_2_. Percent increases (PI) for O_3_ and NO_2_ were 5.5 and 11.3%, respectively.

**Table 2:** Negative binomial regression coefficients (standard errors) for exposure to the pollutants ozone (O_3_), nitrogen dioxide (NO_2_), and particulate matter (PM_10_) according to lags from 0 (LAG 0) up to 7 days (LAG 7) in São José dos Campos, 2020-2021.

	O_3_	NO_2_	PM _10_
LAG 0	**0.005518 (0.002210)**	0.006337 (0.003705)	0.004343 (0.006744)
LAG 1	**0.005913 (0.002195)**	**0.008101 (0 .003654)**	0.002958 (0.006544)
LAG 2	0.004148 (0.002221)	**0.010119 (0.003625)**	0.000305 (0.006566)
LAG 3	0.003865 (0.002144)	**0.011746 (0.003525)**	0.002486 (0.006494)
LAG 4	0.003449 (0.002196)	0.005891 (0.003645)	0.004896 (0.006584)
LAG 5	**0.004569 (0.002161)**	0.006155 (0.003547)	0.007033 (0.006445)
LAG 6	0.003807 (0.002177)	**0.010195 (0.003638)**	-0.001729 (0.006745
LAG 7	**0.005424 (0.002230)**	**0.007020 (0.003579)**	0.000205 (0.000556)

Values in bold type indicate P<0.05.

**Figure 2:**
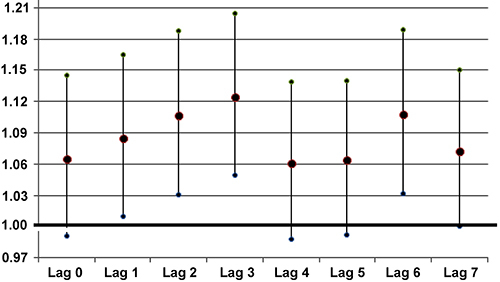
Relative risks (RR) and corresponding 95% confidence intervals for hospitalization for all time lags after a 10 µg/m^3^ increase in O_3_ concentration in the air in São José dos Campos, Brazil in 2020-2021.

**Figure 3:**
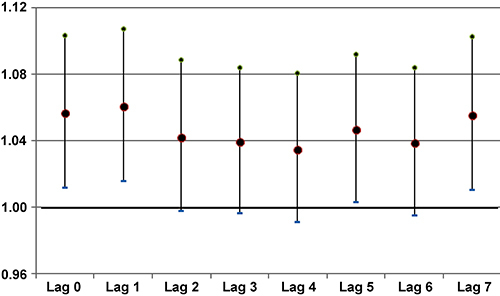
Increased relative risks (RR) and corresponding 95% confidence intervals for hospitalization for all time lags after an increase of 10 µg/m^3^ in the concentration of NO_2_ in the air of São José dos Campos, Brazil in 2020-2021.

Figures 2 and 3 show the graphical representation of RR with the respective 95% confidence intervals for the pollutants for O_3_ and NO_2_.

## Discussion

To the best of our knowledge, this is the first study on a possible correlation between exposure to air pollutants and hospitalization due to COVID-19 in a medium-sized city in Brazil. Ozone and nitrogen dioxide exposures were significant for cases requiring hospitalization, indicating an increased relative risk when exposure occurred two and three days before hospitalization, respectively.

The distribution of cases over the 13 months of the study showed three peaks: July-August 2020, January 2021, and April 2021. These may have been new disease waves or the result of reduced compliance with recommended sanitary measures, such as wearing masks and social distancing, since this municipality did not impose lockdown measures restricting the movement of people.

The cumulative incidence rate found in the municipality was lower than that found for the state of São Paulo of 858.56/per 100,000 inhabitants for the same study period (20).

Hospitalization and death rates showed a predominance of males, as was evidenced by other research (20-23). Female sex proved to have a protective factor against death. Jin et al. (21) had previously studied SARS-CoV's mechanism of cellular entry and found a similar mechanism in SARS-CoV-2, through the angiotensin-converting enzyme 2 receptors (ACE2). Higher protein expression of ACE2 receptors in specific organs and higher levels of circulating ACE2 were observed in men than in women. This has previously been noted in patients with diabetes and cardiovascular diseases (21).

In a national study, Peres et al. (22) observed an average age of 61 years for cases involving hospitalization, for both sexes. The average age for case fatality found in our study was very similar to the mean of 67.1 recorded for the state of São Paulo (20).

Studies show that 74.8% (21) of case fatalities in the state of São Paulo and 85% (22) of hospitalized patients in Brazil had a previous record of at least one health risk condition, with cardiovascular disease being the most frequent. However, this information is not made available through DATASUS.

In a study conducted in Italy (18), the authors identified that in the northern region, which includes Piedmont, Lombardy, Veneto, and Emilia-Romagna, where the highest concentrations of atmospheric pollutants were distributed, there was, among other findings, a clustering of COVID-19 cases with significant correlations. This suggests that the chronic exposure to atmospheric contamination plays a role in the dissemination and virulence of SARS-CoV-2 within a population with a higher incidence of respiratory and cardiac diseases.

Unlike other studies, such as those of Fattorini and Regoli (12) and Khorsandi et al. (24), which found an association between exposure to PM_2.5_ and PM_10_ and high temperatures, hospital admissions, and COVID-19 death rate, our study did not show this association with COVID-19 patient hospitalization.

Increased pulmonary epithelial permeability to the pathogen and pro-inflammatory mediators are mechanisms through which exposure to pollutants could promote SARS-CoV-2 infection. Several studies have shown that exposure to O_3_ and NO_2_ is associated with increased lung permeability due to altered tight junctions, resulting in neutrophil infiltration of the lungs (25).

Evidence suggests that SARS-CoV-2 uses the ACE2 receptor for cell entry in synergy with host transmembrane serine protease 2 (TMPRSS2). More specifically, the viral S-glycoprotein is cleaved by TMPRSS2, which facilitates viral activation and thus represents one of the essential host factors for SARS-CoV-2 pathogenicity (26).

It is well known that NO_2_ can be used as a disinfectant, inactivating both enveloped and non-enveloped viruses, and exposure to O_3_ has been shown to reduce the infectivity of a variety of viruses due to lipid and protein peroxidation. Direct exposure of SARS-CoV-2 to O_3_ likely inactivates enveloped viruses. However, studies with influenza A virus have shown that exposure of human nasal epithelial cells to O_3_ 24 h before infection resulted in increased virus entry and replication, as well as increased levels of secreted TMPRSS2 (26).

Exposure to pollutants may contribute to inflammation and exacerbate SARS-CoV-2-induced lung damage due to increased immune cell infiltration. Studies show that NO_2_ exposure stimulates the release of interleukin (IL)-8, tumor necrosis factor (TNF)-α, and IL-1β from human bronchial epithelial cells. NO_2_ exposure also promotes the adhesion of neutrophils to exposed airway epithelial cells. Experimental exposure of humans to NO_2_ was shown to increase susceptibility to influenza infection and susceptibility of airway epithelial cells to respiratory syncytial virus injury (25).

Exploring environmental factors associated with prevalence and transmission may improve the understanding of COVID-19 and contribute to long-term control strategies.

This study had limitations, among which are those associated with its ecological model. The main limitations result from the type of information collected, which was from secondary data sources, with lack of information about comorbidities associated with COVID-19, and possible diagnostic errors and address errors. Another possible limitation was that no temperature differences in the city were considered. This study does not allow us to establish causality, only a possible association between exposure to air pollutants and COVID-19 hospitalizations. To the best of our knowledge, there are no data from national studies that can be compared with the relative risk values found in São José dos Campos.

Despite the possible limitations mentioned above, associations were found between exposure to air pollutants and hospitalizations due to air pollutants and COVID-19, which may lead to new research fronts in this field.
